# The role of psychotropics on the associations between extreme temperature and heat-related outcomes among people with mental health conditions: population-based study

**DOI:** 10.1017/S0033291724002824

**Published:** 2024-12

**Authors:** Angel Y. S. Wong, Masao Iwagami, Yuta Taniguchi, Chitose Kawamura, Ai Suzuki, Ian J. Douglas, Krishnan Bhaskaran, Takehiro Sugiyama, Naoaki Kuroda, Dorothea Nitsch, Nanako Tamiya

**Affiliations:** 1 Department of Non-Communicable Disease Epidemiology, London School of Hygiene & Tropical Medicine, London, UK; 2 Department of Health Services Research, Institute of Medicine, University of Tsukuba, Ibaraki, Japan; 3 Department of Health Services Research, Graduate School of Comprehensive Human Sciences, University of Tsukuba, Ibaraki, Japan; 4 Diabetes and Metabolism Information Center, Research Institute, National Center for Global Health and Medicine, Shinjuku, Tokyo, Japan; 5 Health Services Research and Development Center, University of Tsukuba, Tsukuba, Ibaraki, Japan; 6 Health Department, Tsukuba, Ibaraki, Japan; 7 Department of Community Mental Health and Law, National Institute of Mental Health, National Center of Neurology and Psychiatry, Kodaira, Tokyo, Japan

**Keywords:** antidepressants, antipsychotics, delirium, heat-related illness, heatwave, myocardial infarction

## Abstract

**Background:**

The association between heatwave and heat-related outcomes in people with mental health conditions with and without psychotropics was unclear.

**Methods:**

We identified people with severe mental illness (SMI) and depression, respectively, using Japanese claim data of Ibaraki prefecture during 1/1/2014–31/12/2021. We conducted self-controlled case series to estimate the incidence rate ratio (IRR) of heat-related illness, myocardial infarction and delirium, respectively, during 5-day pre-heatwave, heatwave, and 5-day post-heatwave periods *v.* all other periods (baseline) within an individual, stratified by periods prescribed psychotropics and periods not prescribed psychotropics, respectively.

**Results:**

Among people with SMI, heatwave was associated with an increased rate of heat-related illness *v.* baseline, with no evidence of a difference in the IRRs between those prescribed *v.* not prescribed antipsychotics (IRR: 1.48 [95% CI 1.40–1.56]; 1.45 [95% CI 1.35–1.56] respectively, *p* interaction: 0.53). Among people with depression, heatwave was similarly associated with heat-related illness, with no evidence of a difference in the IRRs between those prescribed *v.* not prescribed antidepressants (IRR: 1.54 [95% CI 1.46–1.64]; 1.64 [95% CI 1.57–1.71] respectively, *p* interaction: 0.33). Smaller increased rates of heat-related illness were also observed in pre- and post-heatwave periods, *v.* baseline in both cohorts. There was weak evidence of an increased risk of MI and delirium associated with heatwave *v.* baseline.

**Conclusions:**

We showed an increased risk of heat-related illness, myocardial infarction and delirium associated with heatwave in people with mental health conditions regardless of whether being prescribed psychotropics. Risks of heat-related illness, myocardial infarction and delirium associated with heatwave might not be factors to influence decisions about the routine use of psychotropics.

## Introduction

Heatwaves occur more frequently and have lasted longer in recent years. Current evidence suggests that high ambient temperature increases the risk of heat-related mortality (Ban et al., 2017). Previous studies have shown that heatstroke poses negative effects on both the central nervous and cardiovascular system, including increased risk of delirium and myocardial infarction (Ban et al., 2017; Ebi, Exuzides, Lau, Kelsh, & Barnston, 2004; Epstein & Yanovich, 2019; Xu et al., 2023). Increasing temperature was associated with increased risk of mental health hospitalization in people with mental health conditions (Hansen et al., 2008; Sung, Chen, & Su, 2013).

Individuals with psychiatric disorders often require psychotropic medications, including antipsychotics and antidepressants. They can interfere with normal thermoregulatory functions, thereby increasing the risk of heat-related health issues (Ban et al., 2017; Westaway et al., 2015). The use of antipsychotics was associated with an increased risk of hyperthermia or heat stroke during heatwaves, particularly among the elderly (Martin-Latry et al., 2007). To date, studies have not distinguished between the association of mental health condition with heat-related outcomes as opposed to the effect of psychotropic drugs in those with mental health conditions. Further, existing studies have primarily focused on the overall effect of psychotropics, but the associations for specific psychotropics have not been systematically investigated. We therefore hypothesized that there are differences in risk of heat-related outcomes between those with and without psychotropics, and the risks of outcomes varied between types of psychotropics in people with mental health conditions.

In this study, we employed self-controlled case series (SCCS) to examine the associations between extreme heat and heat-related clinical outcomes (i.e. disorders of fluid electrolyte and acid–base balance, and heatstroke; myocardial infarction; delirium) with or without psychotropic medications among individuals with mental health conditions. We also aimed to investigate how these associations differ between individual antipsychotics and antidepressants.

## Methods

### Data source

We used Japanese claim data of Ibaraki prefecture. With the Japanese health insurance system (Matsuda, 2020), we used claims data between 1/1/2014 and 31/12/2021 from the National Health Insurance in Ibaraki prefecture. The National Health Insurance covered approximately 700 thousand individuals (mainly for people aged <75 years who are self-employed or farmers or fishermen, or without regular employment) in Ibaraki prefecture in 2020 (Ibaraki Prefectural Government, 2020), and the current claims data contain cumulative total records of approximately 2 million beneficiaries including demographics, prescriptions, and diagnoses from primary, secondary, and tertiary care settings. Part of the claims data has been used to study the risk of mortality in people with mental health conditions (Kuroda & Tamiya, 2023). We used publicly available meteorological data in Japan from Japan Meteorological Agency (https://www.data.jma.go.jp/risk/obsdl/index.php) to determine the daily mean temperature during the study period (1/1/2014–31/12/2021).

### Study population

We included two study populations: people aged 18–74 years with (1) severe mental illness (SMI) and (2) depression, respectively. We identified mental health conditions using the International Classification of Diseases 10th Revision-10 (ICD-10) (online Supplementary Table S1).

### Study design

We used the SCCS as it compares the incidence rates during pre-defined risk periods with a baseline period within the same individual (Whitaker, Farrington, Spiessens, & Musonda, 2006). We also used case-crossover study design to compare the SCCS findings. Case-crossover study design is also a within-person study design, comparing the odds of exposure in the pre-defined hazard periods with the control periods within an individual (Lewer, Petersen, & Maclure, 2022). The design assumes the underlying population risk of the outcome is not changing substantially over the study period, but does not assume the outcome is independent of the observation period. It therefore serves as a complementary self-controlled design to the SCCS.

### Linked meteorological data

A total of 44 municipalities are geographically located in five major regions of Ibaraki prefecture. We linked the data on mean daily temperatures of six municipalities available from Japan Meteorological Agency to individual data by city code. We selected six municipalities (Kitaibaraki, Hitachi, Mito, Kashima, Tsukuba, and Shimotsuma) which represent the five major regions in Ibaraki prefecture (online Supplementary Fig. S1). Notably, the landscape of the northern region is mostly made up of mountains and wilderness that is different from other municipalities in other regions. Therefore, unlike the other four major regions, two municipalities (Kitaibaraki and Hitachi) were selected in the northern region of Ibaraki.

### Exposure and outcome

For exposure of interest, we defined extreme temperature (i.e. heatwave) as at least 2 consecutive days with daily mean temperature exceeding 95% percentile of that particular year-round, using the meteorological data (Guo et al., 2018).

Primary outcome was heat-related illness (including disorders of fluid electrolyte and acid–base balance, and heatstroke). Secondary outcomes were myocardial infarction and delirium. We identified outcomes using ICD-10 (online Supplementary Table S1). We allow multiple event occurrence for each outcome. An incident event for each outcome was defined as at least 30 days apart between diagnoses (on separate days).

### Self-controlled case series

We included people with a diagnosis of mental health conditions and ever had their outcome of interest during the study period. The analyses were separated for each outcome according to two study populations: people with SMI and people with depression, respectively. People who had both conditions were included in both analyses. The observation period started from the latest of people entering the database, the first diagnosis of mental health conditions, study start date (1/1/2014), until the earliest of patient death, leaving the database, and study end date (31/12/2021). In the main analysis, we stratified the person-time by being prescribed psychotropics (i.e. antipsychotics in people with SMI and antidepressants in people with depression) (online Supplementary Fig. S2). This would allow us to estimate the relative risk of outcomes in periods prescribed psychotropics and periods without being prescribed psychotropics. We used World Health Organization-Anatomical Therapeutic Chemical codes to identify drug exposure (antipsychotics: N05A, antidepressants: N06A) (online Supplementary Table S2). Where there were treatment breaks of ⩽30 days, people were assumed to be exposed to psychotropic drugs continuously, accounting for any potential medication stockpiling and non-adherence. If there were treatment breaks of >30 days, we regarded the periods after the 30 days as periods without being prescribed psychotropics.

Within the observation period, we divided the observation period as a 5-day pre-heatwave, heatwave, and 5-day post-heatwave and all other periods (baseline). We chose to add pre- and post-heatwave periods as this can detect if there was any difference in risk in the lead up to a heatwave and shortly after the heatwave.

To investigate the effect of individual psychotropic drug on clinical outcomes associated with heatwave, we also stratified psychotropics into the five most used antipsychotics and antidepressants, respectively. All other individual psychotropics and those prescribed >1 type of antipsychotics were grouped as one category as ‘other psychotropics’. In this analysis, we started the observation period from entering the database, diagnosis of mental health condition, study start date, or start an antipsychotics/antidepressant prescription and ended the observation period when they left the database, study end date, and discontinuation of the treatment. We also removed people who had psychotropic drugs 180 days before their first prescription identified during the study period to avoid carryover effect of the drug.

### Statistical analysis

The incidence rate ratio (IRR) of each outcome was estimated by comparing the incidence of each outcome between the pre-heatwave/heatwave/post-heatwave period with the baseline period using conditional Poisson regression in people with SMI and depression, respectively. We further controlled for time-varying confounders by adjusting for seasonal effect (Spring: March to May; Summer: June to August; Autumn: September to November; Winter: December to February) and age in a 5-year band. In the main analysis, we introduced the product term of heatwave exposure and prescribed psychotropics as the interaction in the regression to estimate the IRRs for each outcome associated with heatwave among person-time with and without psychotropics, respectively, and performed likelihood ratio test to investigate if the associations differ between person-time with and without psychotropics.

### Subgroup analyses

We studied whether the associations differ by age group and sex in the main analysis. We also further stratified the analysis by use of one psychotropic medication (*v.* multiple psychotropics) in the individual drug analysis. We performed likelihood ratio test to investigate if the associations differ across subgroups.

### Sensitivity analysis/case-crossover study

To test the robustness of the outcome definition, we identified the first outcome during the study period. Second, we excluded people with SMI who only had an inpatient record during the study period, potentially indicating that they were hospitalized for a long time, therefore, unlikely to be exposed to the heatwave. Third, we restricted the observation period to summer seasons only. Fourth, we excluded people diagnosed with both SMI and depression. Fifth, to examine the impact of combined use of antipsychotics and antidepressants, we also adjusted for antidepressant use and antipsychotic use in people with SMI and depression, respectively. Sixth, we excluded people with a prior history of neuroleptic malignant syndrome or serotonin syndrome that could be heat-related.

We used case-crossover study design to compare the SCCS findings as triangulation (online Supplementary Fig. S3). We compared the odds of exposure (occurrence of heatwave) in the hazard period (defined as 5 days) on/prior to event date to the 5-day control period. We also defined a 9-day washout period between the hazard and control period so that the day of the week for the hazard period and control period can be matched. We estimated the odds ratio for each outcome using conditional logistic regression.

Stata/MP 17 was used for data processing and analyses.

### Standard protocol approvals, registrations, and patient consents

The analyses used only de-identified patient-level data, and therefore individual informed consent was not required. The study protocol was approved by the Medical Ethics Committee of the University of Tsukuba in Japan (1845-1) and the London School of Hygiene and Tropical Medicine ethics committee (29904).

## Results

### Description of daily mean temperature

Online Supplementary Fig. S4 shows the plot of daily mean temperatures in six municipalities. Heatwaves occurred with the median of 4–5 times a year. The median of duration of heatwave was 3 days in all municipalities except Tsukuba (4 days) (online Supplementary Table S3). The maximum daily mean temperature during heatwave among all six municipalities ranged from 28.9 to 30.9 °C. During 5-day pre-heatwave and post-heatwave periods, the maximum daily mean temperature was 28.9 °C.

### People with severe mental illness

A total of 62 186 people with SMI were identified during the study period. Of them, 16 534 people experienced heatwave and had ⩾1 diagnosis of heat-related illness (online Supplementary Table S4). Among the person-time not prescribed antipsychotics, the IRR for heat-related illness was 1.45 (95% CI 1.35–1.56) during the heatwave *v.* baseline (Table 1). Similarly, in those prescribed antipsychotics, the IRR was 1.48 (95% CI 1.40–1.56) during the heatwave, *v.* baseline. Increased rates of heat-related illness were also observed in pre- and post-heatwave periods, *v.* baseline but of smaller IRR in contrast to the IRR during heatwave.

**Table 1.** Results of self-controlled case series among people with severe mental illness

	Without antipsychotics				With antipsychotics				
	No. of events	Person-year	IRR	95% CI	No. of events	Person-year	IRR	95% CI	Interaction *p* value for period with and without antipsychotic
Heat-illness									
Baseline	9545	28 422	1 (ref)		18 790	43 261	1 (ref)		0.53
5-day pre-heatwave	410	921	1.12	1.01–1.24	837	1406	1.17	1.09–1.26	
Heatwave	887	1524	1.45	1.35–1.56	1779	2331	1.48	1.40–1.56	
5-day post-heatwave	675	1483	1.15	1.06–1.25	1404	2257	1.23	1.16–1.30	
Myocardial infarction									
Baseline	2780	10 304	1 (ref)		3938	14 573	1 (ref)		0.05
5-day pre-heatwave	80	335	0.86	0.68–1.08	153	474	1.18	1.00–1.40	
Heatwave	185	550	1.20	1.02–1.40	263	783	1.21	1.06–1.39	
5-day post-heatwave	137	535	0.91	0.77–1.09	233	759	1.11	0.96–1.27	
Delirium									
Baseline	304	1727	1 (ref)		768	1692	1 (ref)		0.57
5-day pre-heatwave	12	57	1.09	0.59–1.99	27	55	1.12	0.74–1.68	
Heatwave	15	94	0.81	0.47–1.40	49	92	1.18	0.86–1.64	
5-day post-heatwave	9	92	0.51	0.26–1.01	27	88	0.68	0.45–1.03	

We identified 5277 people who experienced heatwave and had ⩾1 diagnosis of myocardial infarction. Among the person-time not prescribed antipsychotics, the IRR for myocardial infarction was 1.20 (95% CI1.02–1.40) during the heatwave, *v.* baseline (Table 1). Similarly, in those prescribed antipsychotics, the IRR was 1.21 (95% CI 1.06–1.39) during the heatwave, *v.* baseline.

Of 1161 people who experienced heatwave and had ⩾1 diagnosis of delirium, there was no evidence of increased IRRs for delirium regardless of antipsychotic exposure in SCCS (Table 1).

In the individual antipsychotic analysis (online Supplementary Table S5), there was an increased risk of heat-related illness associated with heatwave, *v.* baseline in people prescribed aripiprazole (IRR: 1.56 [95% CI 1.03–2.35]) and sulpiride (IRR: 1.90 [95% CI 1.03–3.48]) respectively. Whilst there was no strong evidence of increased risks in people prescribed risperidone (IRR: 1.26 [95% CI 0.84–1.89]), olanzapine (IRR: 1.36 [95% CI 0.81–2.27]) and quetiapine (IRR: 0.74 [95% CI 0.42–1.31]), the interaction *p* value of 0.26 did not indicate clear differences between individual agents. Power was inadequate for conducting individual antipsychotic analyses for other outcomes.

In the subgroup analyses (online Supplementary Table S6), the highest IRR for heat-related illness during heatwave was observed in people aged 40–49 years (IRR: 1.68 [95% CI 1.54–1.83]; interaction *p* value: 0.01). The IRR of heat-related illness in women was slightly higher than men during post-heatwave period *v.* baseline (interaction *p* value: 0.01). No difference in risk of heat-related illness across subgroups by use of one antipsychotic (*v.* multiple antipsychotics) was observed (online Supplementary Table S7).

### People with depression

A total of 96 875 people with depression were identified during the study period. Of them, 23 645 people experienced heatwave and had ⩾1 diagnosis of heat-related illness (online Supplementary Table S8). Among the person-time not prescribed antidepressants, the IRR was 1.64 (95% CI 1.57–1.71) during the heatwave, *v.* baseline (Table 2). Similarly, in those prescribed antidepressants, the IRR was 1.54 (95% CI 1.46–1.64) during the heatwave, *v.* baseline. Increased rates of heat-related illness were also observed in pre- and post-heatwave periods, *v.* baseline but of smaller IRR in contrast to the IRR during heatwave.

**Table 2.** Results of self-controlled case series among people with depression

	Without antidepressants				With antidepressants				
	No. of events	Person-year	IRR	95% CI	No. of events	Person-year	IRR	95% CI	Interaction *p* value
Heat-illness									
Baseline	23 468	71 342	1 (ref)		14 278	33 063	1 (ref)		0.33
5-day pre-heatwave	1148	2331	1.22	1.15–1.30	706	1078	1.26	1.16–1.36	
Heatwave	2573	3848	1.64	1.57–1.71	1456	1781	1.54	1.46–1.64	
5-day post-heatwave	1845	3737	1.23	1.17–1.30	1082	1720	1.21	1.14–1.29	
Myocardial infarction									
Baseline	8647	30 797	1 (ref)		4267	13 358	1 (ref)		0.67
5-day pre-heatwave	310	1005	1.06	0.94–1.19	148	437	1.03	0.87–1.22	
Heatwave	533	1656	1.10	1.00–1.21	273	716	1.14	1.01–1.30	
5-day post-heatwave	425	1609	0.90	0.81–1.00	228	694	0.99	0.86–1.14	
Delirium									
Baseline	572	2301	1 (ref)		291	966	1 (ref)		0.56
5-day pre-heatwave	20	76	0.95	0.59–1.51	7	31	0.70	0.33–1.51	
Heatwave	32	125	0.93	0.63–1.36	20	51	1.19	0.73–1.91	
5-day post-heatwave	23	121	0.69	0.45–1.06	15	49	0.96	0.56–1.64	

We identified 9407 people who experienced heatwave and had ⩾1 diagnosis of myocardial infarction. Among the person-time not prescribed antidepressants, the IRR was 1.10 (95% CI 1.00–1.21) during the heatwave, *v.* baseline (Table 2). Similarly, in those prescribed antidepressants, the IRR was 1.14 (95% CI 1.01–1.30) during the heatwave, *v.* baseline.

Of 893 people who experienced heatwave and had ⩾1 diagnosis of delirium, there was no evidence of increased IRRs for delirium regardless of being prescribed antidepressants (Table 2).

In the individual antidepressant analysis (online Supplementary Table S9), there was evidence of an increased risk of heat-related illness associated with heatwave, *v.* baseline in people prescribed paroxetine (IRR: 1.65 [95% CI 1.21–2.23]), mirtazapine (IRR: 1.72 [95% CI 1.31–2.25]) and escitalopram (IRR: 1.51 [95% CI 1.35–1.68]) but no strong evidence of increased risks in people prescribed duloxetine (IRR: 1.21 [95% CI 0.90–1.62]) and sertraline (IRR: 1.24 [95% CI 0.85–1.79]; interaction *p* value: 0.11). Power was inadequate for conducting individual antidepressant analysis for other outcomes.

In the subgroup analysis, there was evidence of a higher risk of myocardial infarction associated with heatwave, compared with baseline in men (IRR: 1.22 [95% CI 1.10–1.36]) but not in women (IRR: 1.02 [95% CI 0.91–1.13]; interaction *p* value: 0.05) (online Supplementary Table S10). No difference in risk of heat-related illness across subgroups by use of one antidepressant (*v.* multiple antidepressants) was observed (online Supplementary Table S11).

### Sensitivity analysis/case-crossover study

Results of sensitivity analyses were largely similar to the main analysis. Among people with SMI, case-crossover study showed weak evidence of an increased OR for heat-related illness of 1.29 (95% CI 0.93–1.79) in people without antipsychotics and 1.36 (95% CI 1.08–1.70) in those with antipsychotics. There was no evidence of increased ORs for myocardial infarction regardless of being prescribed antipsychotics. The power was inadequate to conduct case-crossover study for delirium. After adjusting for antidepressant use, we observed a marginal increased risk of myocardial infarction during 5-day post-heatwave in people with SMI and antipsychotic use but not in those without antipsychotics, with the interaction *p* value of 0.02 (online Supplementary Table S12).

Among people with depression, case-crossover study showed an OR for heat-related illness of 1.28 (95% CI 1.10–1.49) in people without antidepressants and 1.44 (95% CI 1.06–1.96) in those with antidepressants. There was no evidence of increased ORs for myocardial infarction and delirium, respectively, regardless of being prescribed antidepressants. After adjusting for antipsychotic use, we observed an increased risk of delirium during heatwave with and without antidepressant use with an interaction *p* value of 0.23 (online Supplementary Table S13).

## Discussion

### Summary of findings

In this population-based study, we observed a 40–60% increased risk of heat-related illness associated with heatwave in people with mental health conditions regardless of whether they were prescribed psychotropic drugs. There was some evidence of an increased risk of myocardial infarction associated with heatwave. There was evidence of an increased risk of delirium associated with heatwave in people with depression regardless of whether they were prescribed antidepressants, after considering the concurrent use of antipsychotics. There was no evidence that the risk of heat-related illness associated with heatwave differed between individual antipsychotics and antidepressants respectively, but the power was limited.

### Findings in context

Similar to our study, a systematic review showed a pooled relative risk (RR) of 1.45 for heat-illness corresponding to change per 1 °C increase in temperature in the general population (Faurie, Varghese, Liu, & Bi, 2022). In a Belgian study, heatwaves were associated with heatstroke (OR: 3.93 on the day of heatstroke; Alsaiqali et al., 2022). In contrast, there was no evidence of an increased risk of myocardial infarction associated with heatwave in the general population (Alsaiqali et al., 2022). However, an increased risk of hospital admissions for myocardial infarction was observed in the South-Central Coast region of Vietnam at extreme high temperatures (Thu Dang et al., 2019). A UK study showed that the risk of myocardial infarction increased by 1.9% for each 1 °C increase in temperature above 20 °C in the general population (Bhaskaran et al., 2012). Another German study showed weak evidence of an increased risk of myocardial infarction associated with heat during 2001–2014 (Chen et al., 2019). Some studies showed that people with SMI were more likely to have dysregulation of body temperature and higher risk of death during heatwaves (Bouchama et al., 2007; Meadows et al., 2024) but the use of psychotropics could also explain the results (Chong & Castle, 2004; Hermesh et al., 2000). Two reviews reported that psychotropics may alter central thermoregulation and impair sweating that may explain their possible effect on heat-related illness (Bongers, Salahudeen, & Peterson, 2020; Westaway et al., 2015). In line with our study, an Australian study showed an increased risk for hospitalization for heat-related illness for 12 months after starting antipsychotics and antidepressants, respectively (Kalisch Ellett, Pratt, Le Blanc, Westaway, & Roughead, 2016). Another French study showed an increased OR of 4.6 for hyperthermia or heatstroke associated with antipsychotics (Martin-Latry et al., 2007). A US study showed that antipsychotics could lead to a higher risk of heat-related hospitalizations during heatwave periods. However, regardless of experiencing heatwave, an increased risk for heat-related hospitalizations was also observed (Layton et al., 2020). Unlike our study, the US study did not investigate the effect of heat-related outcomes for those not prescribed antipsychotics in people with mental health conditions. Importantly, our study found that there was no synergistic effect of antipsychotics/antidepressants on clinical outcomes associated with heatwave among people with mental health conditions in the main analysis. It might indicate that risks of heat-related illness, myocardial infarction and delirium associated with heatwave might not be factors to influence decisions about the routine therapeutic use of psychotropics. However, after adjusting for antidepressants, we showed weak evidence of increased risk of myocardial infarction associated with 5-day post-heatwave in people with SMI and antipsychotics but not those without antipsychotics. However, such pattern was not seen during heatwave in people with SMI nor in people with depression with and without antidepressants. Future study is required to understand the effect of heat on myocardial infarction shortly after heatwave in people with SMI.

To our knowledge, no population-based studies investigated the effects of individual psychotropics on clinical outcomes during heatwaves. Several case reports or spontaneous case reports showed specific psychotropics may lead to hyperthermia or hypothermia (Kao & Kelly, 2007; Kudoh, Takase, & Takazawa, 2003; Lee, Chen, & Chang, 2015; Szota & Araszkiewicz, 2019; van Marum, Wegewijs, Loonen, & Beers, 2007; Zonnenberg, Bueno-de-Mesquita, Ramlal, & Blom, 2017). The direction or degree of thermoregulation could vary between individual psychotropic drugs (Anwer, Abbas, & Saleem, 2014). Although our interaction test showed no evidence that the risk of heat-related illness varied across individual drugs, future studies of larger cohort size would be needed to further investigate the effect of other individual psychotropic drugs including selective serotonin reuptake inhibitors, tricyclic antidepressants, and first-generation antipsychotics (Cheshire & Fealey, 2008).

### Strengths and limitations

This is the first population-based study to study the effect of psychotropics on several clinical outcomes associated with heatwaves and investigate the effect of individual psychotropics on heat-related outcomes. Additionally, the extensive data available enable us to investigate potential effect modifications including age, sex, and concurrent use of other psychotropics. The utilization of the SCCS and case-crossover study designs also eliminate time-invariant confounding.

However, using electronic records to identify drug exposure introduced the possibility of misclassification bias, as we cannot ascertain whether patients took the prescribed drugs. To minimize this bias, we assumed continuous exposure for treatment breaks of ⩽30 days. Further, even though clinical diagnoses in Japan are made according to international criteria (i.e. ICD-10), the validity of the recorded psychiatric diagnoses in the claims data has not yet been assessed. However, the accuracy of the claims data is regularly checked by the insurers. If there are inconsistencies between recorded diagnoses and treatments, the medical institutions cannot receive reimbursement from the insurers. Second, we cannot eliminate residual confounding. Results from subgroup analyses should be interpreted with cautious as they might be impacted by type I error, leading to statistical significance. Moreover, as we used claims data to define outcome, there may be misclassification of outcome if the patient did not see the doctors for their condition and therefore affect the power of the SCCS assuming a non-differential misclassification bias of outcomes during heatwave and baseline. Further, as the data of their outdoor physical activity, housing, and the availability of air-conditioner at home are not available, misclassification of exposure to heatwave is possible. Due to the lack of temperature daily data on all municipalities and definition of heatwave, it could also lead to misclassification of exposure. Notably, we added pre- and post-heatwave periods so that we are still able to capture the risk of events shortly before and after the defined heatwave period. Notably, as the climate and temperature variations are different between regions in Japan and countries, the generalizability of our results may be limited. Further research is required in different regions/countries to confirm the findings. Our study cohort only focused on SMI and depression, the role of antipsychotics/antidepressants in heat-related outcomes during heatwave among people with other mental health conditions, for example, autism spectrum disorders could be explored in future studies.

## Conclusion

We showed that a 40–60% increased risk of heat-related illness associated with heatwave in people with mental health conditions regardless of whether being prescribed psychotropics. There was also evidence of an increased risk of myocardial infarction and delirium associated with heatwave regardless of psychotropics.

## Supporting information

Wong et al. supplementary material Wong et al. supplementary material

## Data Availability

The study data cannot be made available to other researchers because of the terms specified in Data Use Agreements.

## Supplementary material

The supplementary material for this article can be found at https://doi.org/10.1017/S0033291724002824

## Data sharing

Computing code and study protocol are available on GitHub: https://github.com/ehr-lshtm/Heatwave_mental_health.
