# Supplementary material for: The role of psychotropics on the associations between extreme temperature and heat-related outcomes among people with mental health conditions: population-based study
Source: Psychol Med. 2024 Dec 9;54(16):4658–64. doi: 10.1017/S0033291724002824 (PMC11769904; doi:10.1017/S0033291724002824)
Supplement: Wong et al. supplementary material [file S0033291724002824sup001.docx]

**Supplementary material: The role of psychotropics on the associations between extreme temperature and heat-related outcomes in people with mental health conditions: population-based study**

Contents

[Table S1. Diagnostic codes for identifying study population and outcomes 2](#_Toc174023696)

[Table S2. List of antipsychotic and antidepressant prescriptions identified in the Japanese claim data of Ibaraki prefecture using WHO-ATC of N05A and N06A between 2014-2021. 3](#_Toc174023697)

[Figure S1. Linked meteorological data in Ibaraki prefecture 4](#_Toc174023698)

[Figure S2. Illustration of the self-controlled case series design 5](#_Toc174023699)

[Figure S3. Illustration of the case-crossover design 6](#_Toc174023700)

[Figure S4. daily mean temperatures in six municipalities 7](#_Toc174023701)

[Table S3. Description of daily mean temperature in Degree Celsius 8](#_Toc174023702)

[Table S4. Baseline characteristics of people with severe mental illness for each outcome in self-controlled case series 9](#_Toc174023703)

[Table S5. Effects of individual antipsychotics on heat-related illness associated with heatwave in self-controlled case series analysis among people with severe mental illness 10](#_Toc174023704)

[Table S6. Stratified self-controlled case series analysis by age groups and sex among people with severe mental illness 11](#_Toc174023705)

[Table S7. Stratified self-controlled case series analysis by concurrent use of antipsychotics among people with severe mental illness using individual antipsychotic analysis 13](#_Toc174023706)

[Table S8. Baseline characteristics of people with depression for each outcome in self-controlled case series 14](#_Toc174023707)

[Table S9. Effects of individual antidepressants on heat-related illness associated with heatwave in self-controlled case series analysis among people with depression 15](#_Toc174023708)

[Table S10. Stratified self-controlled case series analysis by age groups and sex among people with depression 16](#_Toc174023709)

[Table S11. Stratified self-controlled case series analysis by concurrent use of antidepressants among people with depression using individual antidepressant analysis. 18](#_Toc174023710)

[Table S12. Sensitivity analysis adjusting for use of antidepressants among people with severe mental illness 19](#_Toc174023711)

[Table S13. Sensitivity analysis adjusting for use of antipsychotics among people with depression 20](#_Toc174023712)

Table S1. Diagnostic codes for identifying study population and outcomes

| Study population | International Classification of Diseases 10th Revision-10 (ICD-10) |
| --- | --- |
| Severe mental illness | F20, schizophrenia  F21, schizotypal disorder  F22, persistent delusional disorders  F25, schizoaffective disorders  F30, manic episode  F31, bipolar affective disorder |
| Depression | F32, depressive episode  F33, recurrent depressive disorder |
| Outcome |  |
| Heat-related illness | T67, heatstroke and sunstroke  E86, volume depletion  E87, other disorders of fluid, electrolyte and acid-base balance  X30, exposure to excessive natural heat |
| Myocardial infarction | I21, acute myocardial infarction  I22, subsequent myocardial infarction  I23, certain current complications following acute myocardial infarction |
| Delirium | F05, delirium, not induced by alcohol and other psychoactive substances  R40.0, somnolence  R41.0, disorientation, unspecified |

Table S2. List of antipsychotic and antidepressant prescriptions identified in the Japanese claim data of Ibaraki prefecture using WHO-ATC of N05A and N06A between 2014-2021.

| Psychotropics | | | |
| --- | --- | --- | --- |
| Antipsychotics (N05A) | Drug Name | Antidepressants (N06A) | Drug Name |
| N05AA01 | [chlorpromazine](https://atcddd.fhi.no/atc_ddd_index/?code=N05AA01&showdescription=yes) | N06AX21 | [duloxetine](https://atcddd.fhi.no/atc_ddd_index/?code=N06AX21&showdescription=yes) |
| N05AA02 | [levomepromazine](https://atcddd.fhi.no/atc_ddd_index/?code=N05AA02&showdescription=yes) | N06AB05 | [paroxetine](https://atcddd.fhi.no/atc_ddd_index/?code=N06AB05&showdescription=yes) |
| N05AB02 | [fluphenazine](https://atcddd.fhi.no/atc_ddd_index/?code=N05AB02&showdescription=yes) | N06AX11 | [mirtazapine](https://atcddd.fhi.no/atc_ddd_index/?code=N06AX11&showdescription=yes) |
| N05AB03 | [perphenazine](https://atcddd.fhi.no/atc_ddd_index/?code=N05AB03&showdescription=yes) | N06AX05 | [trazodone](https://atcddd.fhi.no/atc_ddd_index/?code=N06AX05&showdescription=yes) |
| N05AB04 | [prochlorperazine](https://atcddd.fhi.no/atc_ddd_index/?code=N05AB04&showdescription=yes) | N06AB06 | [sertraline](https://atcddd.fhi.no/atc_ddd_index/?code=N06AB06&showdescription=yes) |
| N05AC01 | [periciazine](https://atcddd.fhi.no/atc_ddd_index/?code=N05AC01&showdescription=yes) | N06AB08 | [fluvoxamine](https://atcddd.fhi.no/atc_ddd_index/?code=N06AB08&showdescription=yes) |
| N05AD01 | [haloperidol](https://atcddd.fhi.no/atc_ddd_index/?code=N05AD01&showdescription=yes) | N06AB10 | [escitalopram](https://atcddd.fhi.no/atc_ddd_index/?code=N06AB10&showdescription=yes) |
| N05AD05 | [pipamperone](https://atcddd.fhi.no/atc_ddd_index/?code=N05AD05&showdescription=yes) | N06AA09 | [amitriptyline](https://atcddd.fhi.no/atc_ddd_index/?code=N06AA09&showdescription=yes) |
| N05AD06 | [bromperidol](https://atcddd.fhi.no/atc_ddd_index/?code=N05AD06&showdescription=yes) | N06AX03 | [mianserin](https://atcddd.fhi.no/atc_ddd_index/?code=N06AX03&showdescription=yes) |
| N05AD08 | [droperidol](https://atcddd.fhi.no/atc_ddd_index/?code=N05AD08&showdescription=yes) | N06AA04 | [nomifensine](https://atcddd.fhi.no/atc_ddd_index/?code=N06AX04&showdescription=yes) |
| N05AE01 | [oxypertine](https://atcddd.fhi.no/atc_ddd_index/?code=N05AE01&showdescription=yes) | N06AA17 | [amoxapine](https://atcddd.fhi.no/atc_ddd_index/?code=N06AA17&showdescription=yes) |
| N05AE05 | [lurasidone](https://atcddd.fhi.no/atc_ddd_index/?code=N05AE05&showdescription=yes) | N06AX17 | [milnacipran](https://atcddd.fhi.no/atc_ddd_index/?code=N06AX17&showdescription=yes) |
| N05AG02 | [pimozide](https://atcddd.fhi.no/atc_ddd_index/?code=N05AG02&showdescription=yes) | N06AA02 | [imipramine](https://atcddd.fhi.no/atc_ddd_index/?code=N06AA02&showdescription=yes) |
| N05AH02 | [clozapine](https://atcddd.fhi.no/atc_ddd_index/?code=N05AH02&showdescription=yes) | N06AX16 | [venlafaxine](https://atcddd.fhi.no/atc_ddd_index/?code=N06AX16&showdescription=yes) |
| N05AH03 | [olanzapine](https://atcddd.fhi.no/atc_ddd_index/?code=N05AH03&showdescription=yes) | N06AA21 | [maprotiline](https://atcddd.fhi.no/atc_ddd_index/?code=N06AA21&showdescription=yes) |
| N05AH04 | [quetiapine](https://atcddd.fhi.no/atc_ddd_index/?code=N05AH04&showdescription=yes) | N06AA10 | [nortriptyline](https://atcddd.fhi.no/atc_ddd_index/?code=N06AA10&showdescription=yes) |
| N05AH05 | [asenapine](https://atcddd.fhi.no/atc_ddd_index/?code=N05AH05&showdescription=yes) | N06AX26 | [vortioxetine](https://atcddd.fhi.no/atc_ddd_index/?code=N06AX26&showdescription=yes) |
| N05AL01 | [sulpiride](https://atcddd.fhi.no/atc_ddd_index/?code=N05AL01&showdescription=yes) | N06AA16 | [dosulepin](https://atcddd.fhi.no/atc_ddd_index/?code=N06AA16&showdescription=yes) |
| N05AL02 | [sultopride](https://atcddd.fhi.no/atc_ddd_index/?code=N05AL02&showdescription=yes) | N06AA07 | [lofepramine](https://atcddd.fhi.no/atc_ddd_index/?code=N06AA07&showdescription=yes) |
| N05AL03 | [tiapride](https://atcddd.fhi.no/atc_ddd_index/?code=N05AL03&showdescription=yes) | N06AA06 | [trimipramine](https://atcddd.fhi.no/atc_ddd_index/?code=N06AA06&showdescription=yes) |
| N05AN01 | [lithium](https://atcddd.fhi.no/atc_ddd_index/?code=N05AN01&showdescription=yes) |  |  |
| N05AX08 | [risperidone](https://atcddd.fhi.no/atc_ddd_index/?code=N05AX08&showdescription=yes) |  |  |
| N05AX10 | [mosapramine](https://atcddd.fhi.no/atc_ddd_index/?code=N05AX10&showdescription=yes) |  |  |
| N05AX11 | [zotepine](https://atcddd.fhi.no/atc_ddd_index/?code=N05AX11&showdescription=yes) |  |  |
| N05AX12 | [aripiprazole](https://atcddd.fhi.no/atc_ddd_index/?code=N05AX12&showdescription=yes) |  |  |
| N05AX13 | [paliperidone](https://atcddd.fhi.no/atc_ddd_index/?code=N05AX13&showdescription=yes) |  |  |
| N05AX16 | [brexpiprazole](https://atcddd.fhi.no/atc_ddd_index/?code=N05AX16&showdescription=yes) |  |  |

Figure S1. Linked meteorological data in Ibaraki prefecture

**
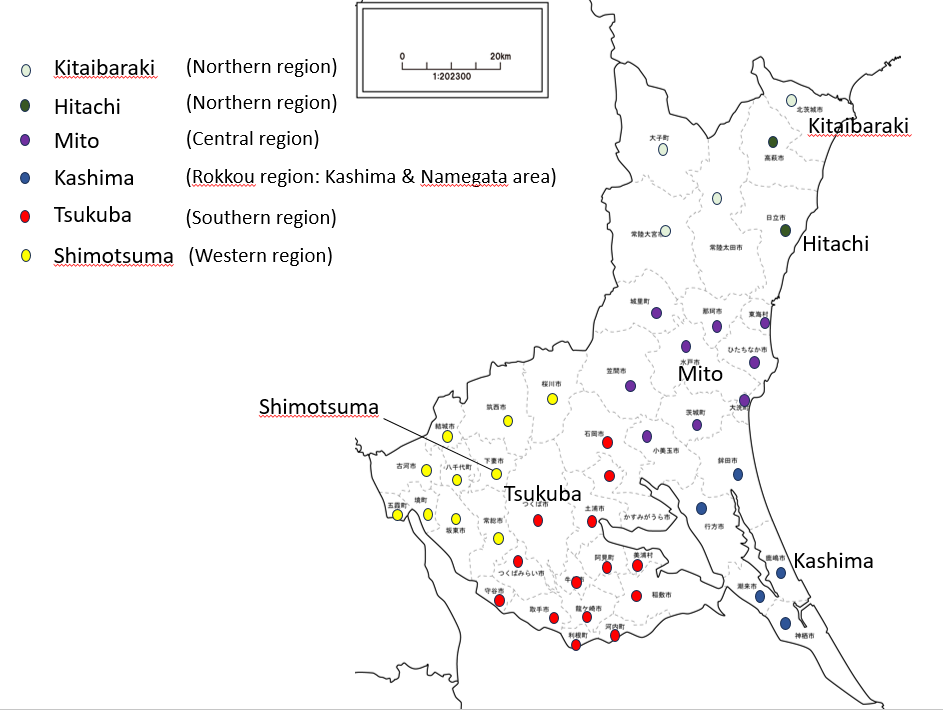
**

Figure S2. Illustration of the self-controlled case series design


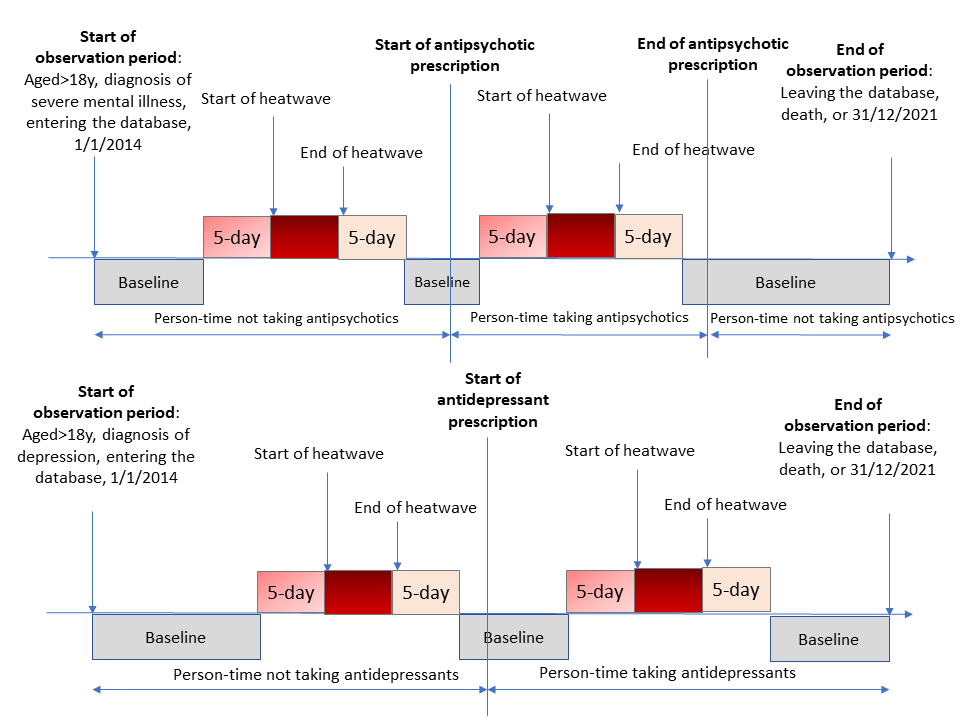


The upper panel illustrates the SCCS analysis for people with severe mental illness; while the lower panel illustrates the SCCS analysis for people with depression

### **Figure S3. Illustration of the case-crossover design**


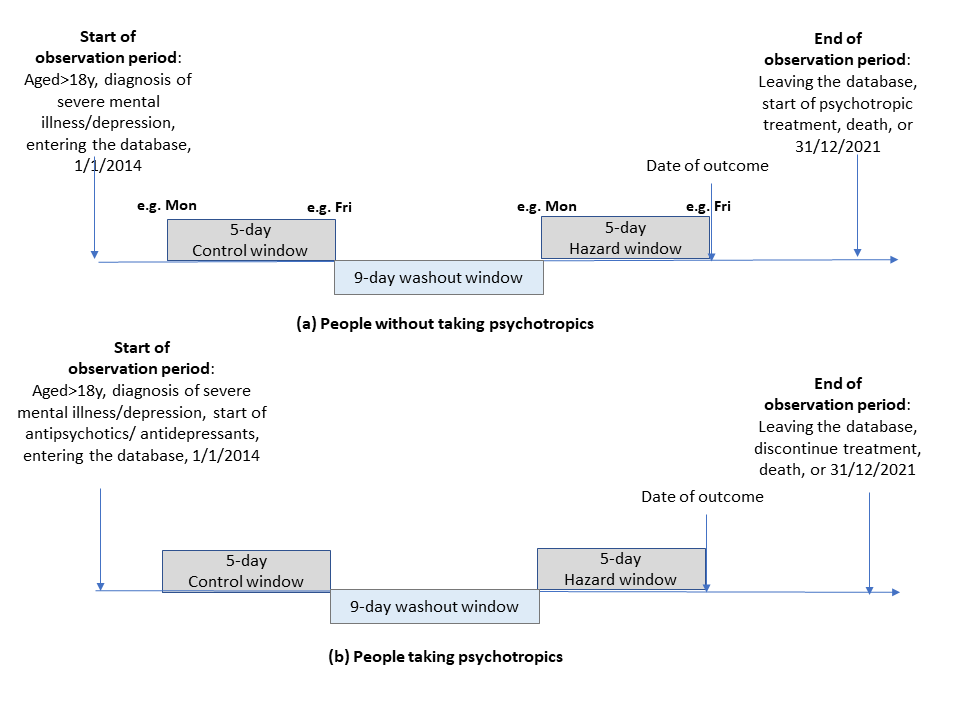


Figure S4. daily mean temperatures in six municipalities


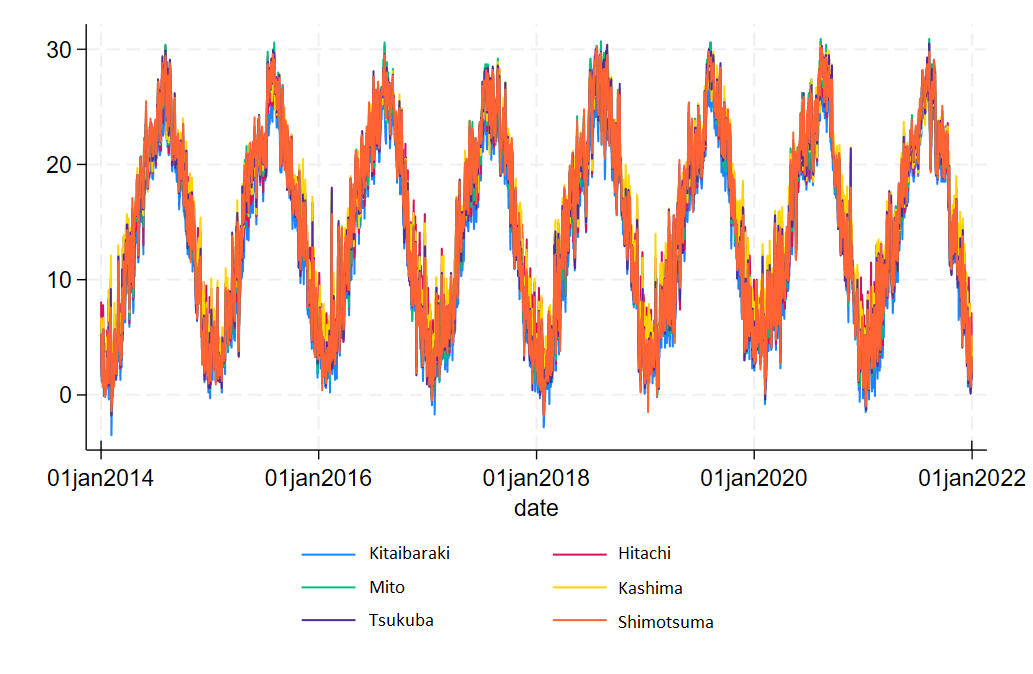


*Vertical axis indicates the temperature in Degree Celsius

Table S3. Description of daily mean temperature in Degree Celsius

|  | Kitaibaraki | Hitachi | Mito | Kashima | Tsukuba | Shimotsuma |
| --- | --- | --- | --- | --- | --- | --- |
| **Pre-heatwave period** |  |  |  |  |  |  |
| Min, Max | 20.5, 26 | 19.3, 28 | 19.6, 28.7 | 19.4, 28.7 | 19, 28,7 | 19.8, 28.9 |
| Median (IQR) | 23.5 (22.1-24.5) | 25.2 (23.6-25.9) | 25.2 (23.9-26.6) | 26.0 (24.6-27) | 25.9 (24.2-27.2) | 25.9 (24.5-27) |
| **Heatwave** |  |  |  |  |  |  |
| Min, Max | 23.6, 28.9 | 25.7, 30.7 | 26.6, 30.9 | 26.3, 30.3 | 26.9, 30.5 | 26.6, 30.2 |
| Median (IQR) |  |  |  |  |  |  |
| Overall (2014-2021) | 25.7 (25.3-26.2) | 27.5 (27-28.3) | 28.7 (27.8-29.2) | 28.4 (27.5-28.8) | 28.5 (28-29.3) | 28.3 (27.6-28.8) |
| 2014 | 25.0 (24.2-25.4) | 26.9 (26.1-27.4) | 28 (27.8-29) | 28.3 (27.7-28.7) | 28.6 (28-28.9) | 28.1 (27.5-28.5) |
| 2015 | 25.5 (24.9-25.8) | 27.8 (27.1-28.7) | 28.8 (27.6-29.5) | 28.5 (27.9-28.8) | 28.7 (28.3-29.2) | 28.5 (27.7-28.8) |
| 2016 | 25.6 (25.3-26) | 27.4 (26.8-28) | 27.4 (26.7-28.6) | 26.9 (26.5-27.9) | 27.3 (27.1-27.8) | 27.3 (27-27.7) |
| 2017 | 24.7 (23.7-25.6) | 26.6 (26.2-27) | 28.4 (27.7-28.6) | 27.6 (27.1-28.3) | 28.2 (27.6-28.3) | 27.5 (27.2-27.9) |
| 2018 | 26.2 (25.6-26.7) | 28.6 (28.1-28.9) | 29.3 (28.9-29.7) | 28.7 (28.6-29.2) | 29.6 (29.4-30) | 29 (28.6-29.5) |
| 2019 | 26 (25.7-26.3) | 27.8 (27.4-28.6) | 29.4 (28.6-29.7) | 28.7 (28.6-29) | 29.2 (28.8-29.6) | 28.8 (28.4-29.2) |
| 2020 | 26.2 (25.8-26.7) | 28.0 (27.7-28.9) | 28.9 (28.4-29.1) | 29.1 (28.6-29.6) | 29 (28.8-29.5) | 29 (28.3-29.3) |
| 2021 | 25.9 (25.7-26.4) | 27.3 (26.9-27.6) | 28.4 (27.6-28.7) | 27.6 (27.1-28.2) | 28.1 (27.8-28.4) | 28 (27.4-28.6) |
| Median (IQR) of duration (in day) | 3 (2-5) | 3 (2-4) | 3 (2-4) | 3 (2-5) | 4 (3-6) | 3 (2-6) |
| **Post-heatwave period** |  |  |  |  |  |  |
| Min, Max | 19, 26.5 | 19.6, 30.1 | 19.5, 30.7 | 20.5, 29.8 | 19.5, 29.8 | 19.3, 28.7 |
| Median (IQR) | 23.5 (22.3-24.2) | 25.1 (23.8-26) | 25.9 (24.4-26.8) | 25.6 (23.9-26.5) | 25.7 (24.5-27) | 25.5 (24.6-26.6) |

*The unit of temperature is shown in °C

Table S4. Baseline characteristics of people with severe mental illness for each outcome in self-controlled case series

|  | Heat-related illness  (n=16,534) | Myocardial infarction  (n=5277) | Delirium  (n=1161) |
| --- | --- | --- | --- |
| Age group |  |  |  |
| 18-<40 | 3994 (24.2) | 975 (18.5) | 72 (6.2) |
| 40-<50 | 2942 (17.8) | 933 (17.7) | 74 (6.4) |
| 50-<60 | 2822 (17.1) | 1011 (19.2) | 132 (11.4) |
| 60-<70 | 4421 (26.7) | 1637 (31.0) | 476 (41.0) |
| ≥70 | 2355 (14.2) | 721 (13.7) | 407 (35.1) |
| Male | 8116 (49.1) | 2722 (51.6) | 739 (63.7) |
|  |  |  |  |
| Comorbidities |  |  |  |
| Ischaemic heart disease | 3680 (22.3) | 1692 (32.1) | 420 (36.2) |
| Cerebrovascular disease | 4569 (27.6) | 1672 (31.7) | 590 (50.8) |
| Diabetes | 9474 (57.3) | 3239 (61.4) | 853 (73.5) |
| Heart failure | 2982 (18.0) | 1264 (24.0) | 402 (34.6) |
| Venous thromboembolism | 1411 (8.5) | 512 (9.7) | 209 (18.0) |
| Hypertension | 5941 (35.9) | 2286 (43.3) | 680 (58.6) |
| Bipolar disorder | 3043 (18.4) | 951 (18.0) | 102 (8.8) |
| Schizophrenia | 13,762 (83.2) | 4397 (83.3) | 1034 (89.1) |
| Chronic obstructive pulmonary disease | 1654 (10.0) | 608 (11.5) | 200 (17.2) |
| Chronic renal disease | 899 (5.4) | 320 (6.1) | 110 (9.5) |
| Cancer | 5796 (35.1) | 2030 (38.5) | 660 (56.9) |
|  |  |  |  |
| Prescription used in the past 90 days |  |  |  |
| ACEis | 307 (1.9) | 160 (3.0) | 38 (3.3) |
| ARBs | 2166 (13.1) | 865 (16.4) | 254 (21.9) |
| Calcium channel blockers | 2875 (17.4) | 1143 (21.7) | 391 (33.7) |
| Diuretics | 1212 (7.3) | 441 (8.4) | 161 (13.9) |
| Beta-blockers | 909 (5.5) | 456 (8.6) | 109 (9.4) |
| Oral anticoagulants | 400 (2.4) | 182 (3.5) | 84 (7.2) |
| Statins | 1867 (11.3) | 797 (15.1) | 184 (15.9) |
| Antibiotics | 4010 (24.3) | 1287 (24.4) | 447 (38.5) |
| Proton pump inhibitors | 3268 (19.8) | 1184 (22.4) | 412 (35.5) |

*Baseline characteristics were measured on/before observation start date

Abbreviation: ACEis, angiotensin-converting enzyme inhibitors; ARBs, angiotensin receptor blockers

### **Table S5. Effects of individual antipsychotics on heat-related illness associated with heatwave in self-controlled case series analysis among people with severe mental illness**

|  | Aripiprazole  (n=167) | | Risperidone (n=205) | | Olanzapine (n=120) | | Quetiapine (n=138) | | Sulpiride  (n=67) | | Interaction p-value |
| --- | --- | --- | --- | --- | --- | --- | --- | --- | --- | --- | --- |
|  | IRR | 95% CI | IRR | 95% CI | IRR | 95% CI | IRR | 95% CI | IRR | 95% CI |  |
| 5-day  pre-heatwave | 1.49 | 0.87-2.54 | 0.96 | 0.54-1.70 | 1.05 | 0.51-2.16 | 1.53 | 0.89-2.62 | 1.89 | 0.90-4.00 | 0.26 |
| Heatwave | 1.56 | 1.03-2.35 | 1.26 | 0.84-1.89 | 1.36 | 0.81-2.27 | 0.74 | 0.42-1.31 | 1.90 | 1.03-3.48 |  |
| 5-day  post-heatwave | 1.33 | 0.85-2.10 | 0.84 | 0.52-1.38 | 0.65 | 0.32-1.34 | 0.79 | 0.45-1.40 | 1.64 | 0.85-3.14 |  |

### **Table S6. Stratified self-controlled case series analysis by age groups and sex among people with severe mental illness**

| Age (years) | IRR | 95% CI | IRR | 95% CI | IRR | 95% CI | IRR | 95% CI | IRR | 95% CI | Interaction p-value |
| --- | --- | --- | --- | --- | --- | --- | --- | --- | --- | --- | --- |
| **Heat-illness** | 18-39 (n=3994) | | 40-49 (n=2942) | | 50-59 (n=2822) | | 60-69 (n=4421) | | ≥70 (n=2355) | |  |
| 5-day pre-heatwave | 1.14 | 1.02-1.29 | 1.20 | 1.06-1.37 | 1.21 | 1.07-1.38 | 1.13 | 1.01-1.27 | 1.04 | 0.87-1.25 | 0.01 |
| Heatwave | 1.51 | 1.39-1.64 | 1.68 | 1.54-1.83 | 1.37 | 1.25-1.51 | 1.40 | 1.29-1.52 | 1.31 | 1.15-1.49 |  |
| 5-day post-heatwave | 1.20 | 1.09-1.31 | 1.25 | 1.13-1.38 | 1.29 | 1.16-1.42 | 1.18 | 1.07-1.29 | 1.00 | 0.87-1.17 |  |
| **Myocardial infarction** | 18-39 (n=975) | | 40-49 (n=933) | | 50-59 (n=1011) | | 60-69 (n=1637) | | ≥70 (n=721) | |  |
| 5-day pre-heatwave | 1.22 | 0.91-1.64 | 1.05 | 0.76-1.44 | 0.97 | 0.72-1.33 | 1.12 | 0.90-1.41 | 0.70 | 0.45-1.08 | 0.36 |
| Heatwave | 1.16 | 0.91-1.47 | 1.33 | 1.06-1.67 | 1.09 | 0.87-1.38 | 1.15 | 0.96-1.37 | 1.39 | 1.08-1.80 |  |
| 5-day post-heatwave | 1.28 | 1.01-1.61 | 0.94 | 0.72-1.23 | 0.96 | 0.75-1.23 | 0.99 | 0.82-1.20 | 0.97 | 0.72-1.31 |  |
| **Delirium** | 18-39 (n=72) | | 40-49 (n=74) | | 50-59 (n=132) | | 60-69 (n=476) | | ≥70 (n=407) | |  |
| 5-day pre-heatwave | 1.32 | 0.41-4.23 | 2.00 | 0.72-5.54 | 0.65 | 0.20-2.07 | 0.90 | 0.51-1.58 | 1.21 | 0.71-2.06 | 0.11 |
| Heatwave | 0.51 | 0.12-2.11 | 2.65 | 1.30-5.41 | 1.89 | 1.06-3.36 | 0.90 | 0.58-1.41 | 0.73 | 0.43-1.23 |  |
| 5-day post-heatwave | 0.53 | 0.13-2.17 | 1.22 | 0.44-3.39 | 0.70 | 0.28-1.74 | 0.65 | 0.39-1.10 | 0.43 | 0.22-0.85 |  |

| Sex | IRR | 95% CI | IRR | 95% CI | Interaction p-value |
| --- | --- | --- | --- | --- | --- |
| **Heat-illness** | Female (n=8418) | | Male (n=8116) | |  |
| 5-day pre-heatwave | 1.13 | 1.04-1.23 | 1.18 | 1.08-1.28 | 0.01 |
| Heatwave | 1.48 | 1.40-1.57 | 1.45 | 1.36-1.54 |  |
| 5-day post-heatwave | 1.28 | 1.20-1.37 | 1.11 | 1.03-1.19 |  |
| **Myocardial infarction** | Female (n=2555) | | Male (n=2722) | |  |
| 5-day pre-heatwave | 1.07 | 0.82-1.23 | 1.07 | 0.89-1.28 | 0.18 |
| Heatwave | 1.20 | 1.03-1.40 | 1.20 | 1.05-1.38 |  |
| 5-day post-heatwave | 1.15 | 0.99-1.34 | 0.92 | 0.79-1.07 |  |
| **Delirium** | Female (n=422) | | Male (n=739) | |  |
| 5-day pre-heatwave | 1.01 | 0.57-1.79 | 1.09 | 0.72-1.66 | 0.45 |
| Heatwave | 0.89 | 0.56-1.44 | 1.11 | 0.80-1.55 |  |
| 5-day post-heatwave | 0.80 | 0.48-1.32 | 0.50 | 0.31-0.80 |  |

### **Table S7. Stratified self-controlled case series analysis by concurrent use of antipsychotics among people with severe mental illness using individual antipsychotic analysis**

|  | 1 type of antipsychotic  (n=930) | | >1 type of antipsychotics  (n=2459) | | Interaction p-value |
| --- | --- | --- | --- | --- | --- |
|  | IRR | 95% CI | IRR | 95% CI |  |
| **Heat-illness** |  |  |  |  |  |
| 5-day pre-heatwave | 1.26 | 0.99-1.60 | 1.07 | 0.91-1.26 | 0.56 |
| Heatwave | 1.35 | 1.12-1.62 | 1.43 | 1.27-1.61 |  |
| 5-day post-heatwave | 1.05 | 0.85-1.30 | 1.12 | 0.99-1.28 |  |

Table S8. Baseline characteristics of people with depression for each outcome in self-controlled case series

|  | Heat-related illness  (n=23,645) | Myocardial infarction  (n=9407) | Delirium  (n=893) |
| --- | --- | --- | --- |
| Age group |  |  |  |
| 18-<40 | 4819 (20.4) | 1134 (12.1) | 56 (6.3) |
| 40-<50 | 3274 (13.9) | 1189 (12.6) | 68 (7.6) |
| 50-<60 | 3478 (14.7) | 1474 (15.7) | 118 (13.2) |
| 60-<70 | 8185 (34.6) | 3956 (42.1) | 406 (45.5) |
| ≥70 | 3889 (16.5) | 1654 (17.6) | 245 (27.4) |
|  |  |  |  |
| Male | 9244 (39.1) | 4157 (44.2) | 487 (54.5) |
|  |  |  |  |
| Comorbidities |  |  |  |
| Ischaemic heart disease | 6296 (26.6) | 3693 (39.3) | 296 (33.2) |
| Cerebrovascular disease | 7523 (31.8) | 3579 (38.1) | 375 (42.0) |
| Diabetes | 13,969 (59.1) | 6078 (64.6) | 626 (70.1) |
| Heart failure | 4560 (19.3) | 2548 (27.1) | 240 (26.9) |
| Venous thromboembolism | 1812 (7.7) | 994 (10.6) | 120 (13.4) |
| Hypertension | 10,487 (44.4) | 5061 (53.8) | 534 (59.8) |
| Chronic obstructive pulmonary disease | 2519 (10.7) | 1221 (13.0) | 135 (15.1) |
| Chronic renal disease | 1269 (5.4) | 608 (6.5) | 54 (6.1) |
| Cancer | 9993 (42.3) | 4484 (47.7) | 495 (55.4) |
|  |  |  |  |
| Prescription used in the past 90 days |  |  |  |
| ACEis | 492 (2.1) | 283 (3.0) | 27 (3.0) |
| ARBs | 4270 (18.1) | 2142 (22.8) | 190 (21.3) |
| Calcium channel blockers | 5222 (22.1) | 2623 (27.9) | 259 (29.0) |
| Diuretics | 1656 (7.0) | 794 (8.4) | 99 (11.1) |
| Beta-blockers | 1673 (7.1) | 1085 (11.5) | 86 (9.6) |
| Oral anticoagulants | 514 (2.2) | 344 (3.7) | 37 (4.1) |
| Statins | 4112 (17.4) | 2139 (22.7) | 162 (18.1) |
| Antibiotics | 5952 (25.2) | 2350 (25.0) | 271 (30.4) |
| Proton pump inhibitors | 5515 (23.3) | 2637 (28.0) | 295 (33.0) |

*Baseline characteristics were measured on/before observation start date

Abbreviation: ACEis, angiotensin-converting enzyme inhibitors; ARBs, angiotensin receptor blockers

### **Table S9. Effects of individual antidepressants on heat-related illness associated with heatwave in self-controlled case series analysis among people with depression**

|  | Duloxetine  (n=437) | | Sertraline  (n=288) | | Paroxetine (n=300) | | Mirtazapine (n=345) | | Escitalopram  (n=199) | | Interaction p-value |
| --- | --- | --- | --- | --- | --- | --- | --- | --- | --- | --- | --- |
|  | IRR | 95% CI | IRR | 95% CI | IRR | 95% CI | IRR | 95% CI | IRR | 95% CI |  |
| 5-day  pre-heatwave | 1.46 | 1.03-2.05 | 1.31 | 0.82-2.07 | 1.35 | 0.89-2.04 | 1.55 | 1.09-2.22 | 1.20 | 1.04-1.39 | 0.11 |
| Heatwave | 1.21 | 0.90-1.62 | 1.24 | 0.85-1.79 | 1.65 | 1.21-2.23 | 1.72 | 1.31-2.25 | 1.51 | 1.35-1.68 |  |
| 5-day  post-heatwave | 1.30 | 0.97-1.75 | 1.50 | 1.06-2.13 | 0.93 | 0.63-1.38 | 0.77 | 0.52-1.14 | 1.17 | 1.04-1.32 |  |

### **Table S10. Stratified self-controlled case series analysis by age groups and sex among people with depression**

| Age (years) | IRR | 95% CI | IRR | 95% CI | IRR | 95% CI | IRR | 95% CI | IRR | 95% CI | Interaction p-value |
| --- | --- | --- | --- | --- | --- | --- | --- | --- | --- | --- | --- |
|  | 18-39 (n=4819) | | 40-49 (n=3274) | | 50-59 (n=3478) | | 60-69 (n=8185) | | ≥70 (n=3889) | |  |
| **Heat-illness** |  |  |  |  |  |  |  |  |  |  |  |
| 5-day pre-heatwave | 1.26 | 1.14-1.40 | 1.25 | 1.10-1.41 | 1.33 | 1.18-1.49 | 1.20 | 1.10-1.30 | 1.16 | 1.02-1.33 | 0.17 |
| Heatwave | 1.54 | 1.43-1.67 | 1.75 | 1.61-1.91 | 1.62 | 1.49-1.76 | 1.58 | 1.50-1.68 | 1.54 | 1.41-1.69 |  |
| 5-day post-heatwave | 1.13 | 1.03-1.23 | 1.19 | 1.08-1.32 | 1.33 | 1.21-1.46 | 1.24 | 1.16-1.32 | 1.24 | 1.12-1.37 |  |
| **Myocardial infarction** | 18-39 (n=1134) | | 40-49 (n=1189) | | 50-59 (n=1474) | | 60-69 (n=3956) | | ≥70 (n=1654) | |  |
| 5-day pre-heatwave | 0.96 | 0.71-1.30 | 1.39 | 1.09-1.76 | 0.93 | 0.73-1.19 | 1.08 | 0.94-1.24 | 0.88 | 0.68-1.14 | 0.10 |
| Heatwave | 1.03 | 0.82-1.30 | 1.40 | 1.16-1.68 | 1.04 | 0.87-1.24 | 1.06 | 0.94-1.19 | 1.18 | 0.99-1.41 |  |
| 5-day post-heatwave | 1.03 | 0.82-1.30 | 1.04 | 0.84-1.30 | 0.95 | 0.79-1.15 | 0.90 | 0.80-1.02 | 0.83 | 0.67-1.03 |  |
| **Delirium** | 18-39 (n=56) | | 40-49 (n=68) | | 50-59 (n=118) | | 60-69 (n=406) | | ≥70 (n=245) | |  |
| 5-day pre-heatwave | 1.52 | 0.47-4.89 | 1.36 | 0.42-4.37 | 0.25 | 0.03-1.81 | 0.83 | 0.46-1.50 | 0.94 | 0.45-1.94 | 0.28 |
| Heatwave | 0.90 | 0.28-2.89 | 1.89 | 0.86-4.20 | 1.82 | 0.98-3.37 | 0.69 | 0.42-1.15 | 0.97 | 0.54-1.74 |  |
| 5-day post-heatwave | 0.32 | 0.04-2.30 | 1.13 | 0.41-3.14 | 0.47 | 0.15-1.50 | 0.81 | 0.50-1.31 | 0.86 | 0.46-1.60 |  |

| Sex | IRR | 95% CI | IRR | 95% CI | Interaction p-value |
| --- | --- | --- | --- | --- | --- |
| **Heat-illness** | Female (n=14,401) | | Male (n=9244) | |  |
| 5-day pre-heatwave | 1.23 | 1.15-1.31 | 1.24 | 1.15-1.34 | 0.18 |
| Heatwave | 1.58 | 1.51-1.66 | 1.63 | 1.54-1.72 |  |
| 5-day post-heatwave | 1.26 | 1.20-1.32 | 1.16 | 1.09-1.24 |  |
| **Myocardial infarction** | Female (n=5250) | | Male (n=4157) | |  |
| 5-day pre-heatwave | 1.08 | 0.95-1.23 | 1.01 | 0.88-1.17 | 0.05 |
| Heatwave | 1.02 | 0.91-1.13 | 1.22 | 1.10-1.36 |  |
| 5-day post-heatwave | 0.96 | 0.86-1.07 | 0.90 | 0.80-1.02 |  |
| **Delirium** | Female (n=406) | | Male (n=487) | |  |
| 5-day pre-heatwave | 0.74 | 0.40-1.37 | 0.98 | 0.58-1.64 | 0.89 |
| Heatwave | 1.04 | 0.67-1.59 | 0.98 | 0.65-1.49 |  |
| 5-day post-heatwave | 0.73 | 0.44-1.20 | 0.81 | 0.51-1.28 |  |

### **Table S11. Stratified self-controlled case series analysis by concurrent use of antidepressants among people with depression using individual antidepressant analysis.**

|  | 1 type of antidepressant  (n=2197) | | >1 type of antidepressants  (n=2020) | | Interaction p-value |
| --- | --- | --- | --- | --- | --- |
|  | IRR | 95% CI | IRR | 95% CI |  |
| **Heat-illness** |  |  |  |  |  |
| 5-day pre-heatwave | 1.34 | 1.14-1.57 | 1.21 | 1.03-1.43 | 0.75 |
| Heatwave | 1.52 | 1.34-1.72 | 1.47 | 1.29-1.66 |  |
| 5-day post-heatwave | 1.19 | 1.04-1.37 | 1.11 | 0.97-1.28 |  |

### **Table S12. Sensitivity analysis adjusting for use of antidepressants among people with severe mental illness**

|  | Without antipsychotics | | With antipsychotics | | Interaction  p-value |
| --- | --- | --- | --- | --- | --- |
|  | IRR | 95% CI | IRR | 95% CI |  |
| **Heat-illness** |  |  |  |  |  |
| baseline | 1 (ref) |  | 1 (ref) |  | 0.21 |
| 5-day pre-heatwave | 1.16 | 1.05-1.29 | 1.24 | 1.16-1.34 |  |
| Heatwave | 1.50 | 1.39-1.61 | 1.56 | 1.48-1.65 |  |
| 5-day post-heatwave | 1.20 | 1.11-1.30 | 1.30 | 1.23-1.38 |  |
| **Myocardial infarction** |  |  |  |  |  |
| baseline | 1 (ref) |  | 1 (ref) |  | 0.02 |
| 5-day pre-heatwave | 0.88 | 0.70-1.11 | 1.24 | 1.05-1.47 |  |
| Heatwave | 1.23 | 1.05-1.44 | 1.27 | 1.11-1.46 |  |
| 5-day post-heatwave | 0.94 | 0.79-1.13 | 1.17 | 1.01-1.35 |  |
| **Delirium** |  |  |  |  |  |
| baseline | 1 (ref) |  | 1 (ref) |  | 0.51 |
| 5-day pre-heatwave | 1.10 | 0.60-2.02 | 1.17 | 0.77-1.77 |  |
| Heatwave | 0.82 | 0.48-1.41 | 1.22 | 0.89-1.69 |  |
| 5-day post-heatwave | 0.52 | 0.26-1.03 | 0.71 | 0.47-1.07 |  |

### **Table S13. Sensitivity analysis adjusting for use of antipsychotics among people with depression**

|  | Without antipsychotics | | With antipsychotics | | Interaction  p-value |
| --- | --- | --- | --- | --- | --- |
|  | IRR | 95% CI | IRR | 95% CI |  |
| **Heat-illness** |  |  |  |  |  |
| baseline | 1 (ref) |  | 1 (ref) |  | 0.10 |
| 5-day pre-heatwave | 1.33 | 1.25-1.42 | 1.48 | 1.36-1.60 |  |
| Heatwave | 1.78 | 1.70-1.86 | 1.79 | 1.69-1.90 |  |
| 5-day post-heatwave | 1.35 | 1.29-1.42 | 1.44 | 1.35-1.53 |  |
| **Myocardial infarction** |  |  |  |  |  |
| baseline | 1 (ref) |  | 1 (ref) |  | 0.18 |
| 5-day pre-heatwave | 1.12 | 0.99-1.26 | 1.15 | 0.97-1.36 |  |
| Heatwave | 1.15 | 1.05-1.27 | 1.27 | 1.12-1.44 |  |
| 5-day post-heatwave | 0.95 | 0.86-1.06 | 1.12 | 0.97-1.28 |  |
| **Delirium** |  |  |  |  |  |
| baseline | 1 (ref) |  | 1 (ref) |  | 0.23 |
| 5-day pre-heatwave | 1.57 | 0.98-2.52 | 1.47 | 0.67-3.20 |  |
| Heatwave | 1.49 | 1.01-2.20 | 2.39 | 1.46-3.90 |  |
| 5-day post-heatwave | 1.20 | 0.77-1.87 | 2.06 | 1.17-3.61 |  |
